# FDA-Approved Monoclonal Antibodies for Unresectable Hepatocellular Carcinoma: What Do We Know So Far?

**DOI:** 10.3390/ijms24032685

**Published:** 2023-01-31

**Authors:** Iason Psilopatis, Christos Damaskos, Anna Garmpi, Panagiotis Sarantis, Evangelos Koustas, Efstathios A. Antoniou, Dimitrios Dimitroulis, Gregory Kouraklis, Michail V. Karamouzis, Kleio Vrettou, Georgios Marinos, Konstantinos Kontzoglou, Nikolaos Garmpis

**Affiliations:** 1 Department of Gynecology, Charité-Universitätsmedizin Berlin, Corporate Member of Freie Universität Berlin and Humboldt-Universität zu Berlin, Augustenburger Platz 1, 13353 Berlin, Germany; 2 Renal Transplantation Unit, Laiko General Hospital, 11527 Athens, Greece; 3 N.S. Christeas Laboratory of Experimental Surgery and Surgical Research, Medical School, National and Kapodistrian University of Athens, 11527 Athens, Greece; 4 First Department of Propedeutic Internal Medicine, Laiko General Hospital, Medical School, National and Kapodistrian University of Athens, 11527 Athens, Greece; 5 Molecular Oncology Unit, Department of Biological Chemistry, Medical School, National and Kapodistrian University of Athens, 11527 Athens, Greece; 6 Second Department of Propedeutic Surgery, Laiko General Hospital, Medical School, National and Kapodistrian University of Athens, 11527 Athens, Greece; 7 Department of Cytopathology, Sismanogleio General Hospital, 15126 Athens, Greece; 8 Department of Hygiene, Epidemiology and Medical Statistics, Medical School, National and Kapodistrian University of Athens, 11527 Athens, Greece

**Keywords:** hepatocellular, carcinoma, unresectable, treatment, FDA, monoclonal, antibody

## Abstract

Unresectable hepatocellular carcinoma (HCC) is an advanced primary liver malignancy with a poor prognosis. The Food and Drug Administration (FDA) has, to date, approved nivolumab, pembrolizumab, ramucirumab, nivolumab/ipilimumab, atezolizumab/bevacizumab, as well as tremelimumab/durvalumab, as first- or second-line monoclonal antibodies (mAbs) for unresectable HCC. The present review examines the current state of knowledge, and provides a useful update on the safety and efficacy of these therapeutic agents, thus attempting to define the suitability of each mAb for different patient subgroups.

## 1. Introduction

Hepatocellular carcinoma (HCC) represents the leading form of primary liver cancer in adults. For 2022, the American Cancer Society estimates the incidence of primary liver and intrahepatic bile duct cancer at 41,260 cases and the related deaths at 30,520, in the United States [1]. Hepatic cirrhosis, chronic viral hepatitis B or C, non-alcoholic steatohepatitis (NASH), excessive alcohol consumption, and smoking, as well as aflatoxin ingestion, embody the most common causative factors of HCC [2]. Patients with HCC mostly only show symptoms of the underlying etiological conditions, while signs of advanced HCC might include cancer cachexia, hepatomegaly, jaundice, or ascites [3]. Screening for HCC in at-risk patients routinely involves alpha-fetoprotein (AFP) blood tests and abdominal ultrasound exams every six months [4]. For patients with early-stage resectable HCC, partial hepatectomy or liver transplantation represents the first-line therapy [5,6]. Patients with more advanced stage HCC may be treated with radiofrequency ablation, transcatheter arterial embolization, targeted therapy, immunotherapy, chemotherapy, or radiotherapy [5,7].

Given its aggressiveness, the 5-year survival rate for patients diagnosed with liver cancer even in a localized Surveillance, Epidemiology, and End Results (SEER) stage amounts to only 35%, with the survival rates remaining low for patients with unresectable HCC [8]. Fortunately, the employment of novel efficient targeted drugs seems to signify the beginning of a promising era in the treatment of unresectable HCC.

Following its Food and Drug Administration (FDA) approval in 2007, sorafenib remained the only first-line approved targeted agent for patients with unresectable HCC until 2017 [9]. Sorafenib is a multi-kinase inhibitor that targets various serine/threonine and tyrosine kinases, thus offering tumor cell proliferation and angiogenesis inhibition [10]. In 2017, regorafenib became the second oral multi-kinase inhibitor to receive FDA approval as a second-line systemic drug for unresectable HCC [11]. The same year, nivolumab, a programmed cell death protein 1 (PD-1) inhibitor, became the first FDA-approved monoclonal antibody (mAb) for second-line targeted therapy in the treatment of HCC [12]. mAbs are single, highly-specific immunoglobulins cloned from a single B cell that, by safely targeting specific antigens, deplete cells or block receptor–ligand interactions [13]. In cancer therapy, mAbs may both directly target tumor cells and induce long-lasting anti-cancer immune responses [14]. FDA has, so far, approved a great number of mAbs alone, or in combination with other drugs, as first- or later-line therapeutic regimes for diverse cancer entities [15].

The current review intends to summarize the results of all relevant clinical trials on the FDA-approved mAbs, thus providing a useful update and identifying obscure aspects requiring further research. The literature review was conducted using the MEDLINE and LIVIVO databases. The search terms nivolumab, pembrolizumab, ramucirumab, ipilimumab, atezolizumab, bevacizumab, tremelimumab, durvalumab, and unresectable HCC were employed, and we were able to identify fifty-one relevant studies in English, published between 2018 and 2022.

## 2. FDA-Approved Monoclonal Antibodies for Unresectable Hepatocellular Carcinoma

**Figure 1:**
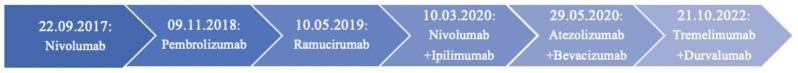
Timeline of FDA-approved monoclonal antibodies for unresectable hepatocellular carcinoma.

After the initial approval of nivolumab in 2017, one year later, pembrolizumab, another PD-1 inhibitor, followed suit [16]. In 2019, the vascular endothelial growth factor receptor 2 (VEGFR2)-antagonist ramucirumab also received FDA approval as a single agent for HCC [17], while, in 2020, the FDA approved another two combinational mAb therapeutic regimens: nivolumab/ipilimumab and atezolizumab/bevacizumab [18,19]. On 21 October 2022, tremelimumab/durvalumab became the most recently FDA-approved combinational mAb therapeutic regimen for adult patients with unresectable HCC [20]. Notably, atezolizumab/bevacizumab nowadays represents the first-line therapy for advanced, treatment-naïve HCC (Figure 1) [21].

### 2.1. Nivolumab

Nivolumab is an immunoglobulin G4 (IgG4) PD-1 immune checkpoint inhibitor antibody, which disrupts the interaction of the PD-1 receptor with its ligands, thereby stimulating a memory response to tumor antigen-specific T cell proliferation [22].

CheckMate 040 is a randomized, multicenter, open-label, phase III trial, comparing nivolumab versus sorafenib monotherapy for patients with advanced HCC [23]. Based on its results, FDA approved nivolumab as a second-line treatment alternative for HCC previously treated with sorafenib [12]. Since its approval, several study groups have specifically investigated the role of nivolumab in the treatment of unresectable HCC. Lee et al. were the first to publish their results on response and survival predictors in nivolumab- or pembrolizumab-treated unresectable HCC. By evaluating the radiologic images of a total of 90 HCC patients according to the revised Response Evaluation Criteria in Solid Tumors (RECIST) 1.1, an objective response rate (ORR) of approximately 25% was reported, with patients at Child–Pugh A or under combinational treatment exhibiting higher ORR. Early AFP reduction represented an independent predictor of OR, as well as of overall survival (OS), together with Child–Pugh class A and Albumin-Bilirubin (ALBI) grade 1 [24]. Similarly, Hsu et al. retrospectively enrolled 87 nivolumab-treated patients with unresectable HCC, and, by employing radiological imaging techniques, described an ORR of ca. 20%. The presence of a single tumor and a significant decline in AFP levels were found to be predictors of OR, while progression-free survival (PFS) directly correlated with macrovascular invasion absence, concurrent therapy, and AFP response. Remarkably, both a low Cancer of the Liver Italian Program (CLIP) score and low-grade immune-related adverse events were associated with a higher OS [25]. Given the seemingly important role of AFP response as a predictor of ORR, PFS, and OS in unresectable HCC, Teng et al. developed a 50-10 rule of AFP responses to predict the prognosis of nivolumab-treated HCC patients, including delayed AFP responders. According to this rule, patients showing a more than 50% decline in AFP level after one month of nivolumab monotherapy will most probably profit from a greater ORR, PFS, or OS, whereas prognosis prediction of patients with AFP change at week 4 within ±50% from baseline requires further AFP level determination at week 12, in order to discriminate nivolumab monotherapy as a first- or second-line therapeutic regime [26]. Furthermore, Lewis et al. retrospectively assessed 58 patients after nivolumab monotherapy for unresectable HCC using RECIST 1.1, modified RECIST, and immune RECIST, and unanimously described an ORR of 21%, thus demonstrating the equivalent performance of standard and immune response criteria for foreseeing OS. Of note, prior transarterial radioembolization (TARE), a minimally invasive treatment option generally proposed to patients with reduced hepatic function and/or greater tumor burden, was identified as the main predictor of HCC progression, which, in turn, correlated with poor OS [27]. Interestingly, a South Korean study group evaluated nivolumab monotherapy against unresectable HCC in a hepatitis B virus (HBV)-endemic region, and also found a similar ORR. Multivariate analysis revealed that small tumor diameters and low ALBI grades significantly correlated with OS [28].

### 2.2. Pembrolizumab

Pembrolizumab is another humanized monoclonal anti-PD-1 antibody that hinders tumor cells from evading anti-tumor immunity [29].

The FDA granted accelerated approval to pembrolizumab for patients with HCC who have been previously treated with sorafenib, based on the results of KEYNOTE-224, a non-randomized, multicenter, open-label, phase II trial [30]. Following this study, numerous research groups studied the efficacy of pembrolizumab in advanced, unresectable HCC. Feun et al. conducted a phase II study of pembrolizumab on 29 patients with unresectable HCC, and reported an ORR of 32%, and a median OS of more than a year, with low transforming growth factor beta (TGF-β) levels significantly correlating with better treatment outcomes [31]. Given the proven efficacy of pembrolizumab in microsatellite instability-high solid tumors [32], Kawaoka et al. investigated the incidence of microsatellite instability-high tumors in a Japanese patient cohort with pretreated, unresectable HCC. However, pembrolizumab treatment was found to be effective in only one of the two patients (2/82) with a positive microsatellite instability-high tumor status, whereas the other patient was a non-responder [33].

Four different study groups also explored the synergistic effects of pembrolizumab/lenvatinib combinational treatment in patients with unresectable HCC, as lenvatinib is a potent FDA-approved receptor tyrosine kinase inhibitor (TKI) for the first-line treatment of patients with unresectable HCC [34]. Finn et al. enrolled a total of 104 patients in a phase Ib study, and described confirmed, durable response rates per an independent imaging review, a median PFS of 9 months by RECIST, as well as a median OS of almost two years [35]. In a similar study incorporating 84 HCC patients, the median PFS and OS were 6.6 and 11.4 months, respectively, with tumor occupation ≥50% volume of liver representing an independent predictor for treatment efficacy and patient survival [36]. Moreover, Wu et al. prospectively enrolled 71 patients with unresectable HCC, and, by performing multivariate analysis, determined Child–Pugh class B and prior nivolumab failure as independent factors for poorer OS [37]. In order to further evaluate pembrolizumab plus lenvatinib with or without transarterial chemoembolization (TACE) for the treatment of unresectable HCC, Chen et al. retrospectively reviewed a large patient collective, and concluded that the promising combination of pembrolizumab plus lenvatinib with TACE is associated with a controllable safety profile, longer survival time, as well as a higher rate of conversion therapy in programmed death-ligand 1 (PD-L1)-positive, treatment-naïve unresectable HCC [38,39].

### 2.3. Ramucirumab

Ramucirumab is a recombinant human IgG1 monoclonal antibody that binds to VEGFR-2 and blocks VEGF-A-stimulated endothelial cell proliferation and migration, hence down-regulating tumor vascularity and growth [40].

The promising results of the randomized, double-blind, placebo-controlled, phase III trial REACH-2 led to the FDA approval of ramucirumab as a single agent for HCC in patients with AFP levels ≥ 400 ng/mL and previously treated with sorafenib [41]. So far, three original articles have been published on the efficacy and safety of ramucirumab in patients with unresectable HCC. Amioka et al. compared the clinical outcome of ramucirumab as a later-line agent in multi-molecular targeted agent sequential therapy for unresectable HCC, and stated that ramucirumab efficiency is determined by the AFP response to ramucirumab, as well as the treatment response to prior TKI therapy [42]. Hiraoka et al. studied the therapeutic efficacy of ramucirumab in 28 patients with lenvatinib-pretreated unresectable HCC, and reported an extremely low ORR of approximately 4%, as well as a disappointing disease-control rate of 42.3% [43]. Analogously, Kasuya et al. evaluated the application of ramucirumab as a second- or third-line treatment in seven patients with lenvatinib-pretreated unresectable HCC, and reported an even lower disease-control rate of almost 29% and a median PFS of 41 days. Notably, the AFP production rate did, nevertheless, decrease from the baseline in patients with stable disease, thus predicting ramucirumab effectiveness [44].

### 2.4. Nivolumab–Ipilimumab

Ipilimumab is a human IgG1 mAb that binds cytotoxic T-lymphocyte antigen-4 (CTLA-4), hence prolonging T-cell activation and restoring T-cell proliferation [45].

After investigating the efficacy of the combination in Cohort 4 of the CheckMate 040 trial, FDA granted accelerated approval to nivolumab/ipilimumab for patients with sorafenib-pretreated HCC [18]. Juloori et al. recently published the results of the first phase I prospective trial on stereotactic body radiation therapy (SBRT) followed by nivolumab/ipilimumab or nivolumab monotherapy in unresectable HCC, and concluded that clinical outcomes favored nivolumab/ipilimumab in terms of ORR (57%), PFS (11.6 months), and OS (41.6 months) [46].

### 2.5. Atezolizumab–Bevacizumab

Atezolizumab is an anti-PD-L1 antibody that reinvigorates cytotoxic T cells to re-express their antitumor effect. Bevacizumab represents an anti-VEGF antibody that not only shows anti-angiogenic effects, but also diminishes the activity of immunosuppressive cells in the tumor microenvironment [47].

Following the publication of the results of the global, open-label, phase III IMbrave150 trial in mid-2020, atezolizumab/bevacizumab became another FDA-approved mAb (combination) for patients with treatment-naïve unresectable or metastatic HCC [48]. The study population of the IMbrave150 trial has, so far, represented the study material for numerous consequent studies. Cheng et al. performed an updated analysis one year after the primary analysis of IMbrave150, and reported an atezolizumab/bevacizumab-associated ORR of 30%, a similar safety profile, as well as a median OS of 5.8 months longer with atezolizumab/bevacizumab than sorafenib [49], while Qin et al. described the results of an extension phase of IMbrave150, and suggested clinically meaningful improvements in OS and PFS in the additionally enrolled Chinese subpopulation, despite the higher HBV infection rate and the poorer prognostic factors of the Asian population [50]. Moreover, Salem et al. conducted a post hoc analysis of ORR, depth of response, and complete response, and found the atezolizumab/bevacizumab treatment to positively correlate with all three parameters [51]. Galle et al. aimed at objectively assessing the subjective symptoms of HCC patients enrolled in the IMbrave150 trial by the use of quality-of-life questionnaires, and underlined the significant improvement in quality of life, functioning, and HCC symptoms in patients treated with atezolizumab/bevacizumab [52]. In terms of cost-effectiveness, four independent study groups also performed analyses based on the IMbrave150 trial, and reached the unanimous conclusion that, despite its promising anticancer potential, atezolizumab/bevacizumab does not represent a cost-effective first-line systemic treatment of unresectable HCC [53,54,55,56].

Additionally, three studies incorporated the patient data from both the IMbrave150 and the phase Ib GO30140 trial. Specifically, Shemesh et al. assessed pharmacokinetics and safety based on hepatic impairment status and geographic region, and suggested no atezolizumab/bevacizumab dose adjustment for mild or moderate hepatic impairment, or specific geographic region [57]. Zhu et al. employed transcriptomic, genetic, in situ multiplex immunohistochemistry in HCC samples from patients enrolled in the above-mentioned trials, and found a direct association of pre-existing immunity in baseline tumors, high PD-L1 mRNA levels, enriched (myeloid) inflammatory response, high CD8+/regulatory T-cell count, and high VEGFR2 expression, with atezolizumab/bevacizumab activity. On the contrary, a high regulatory-to-effector T-cell ratio and oncofetal gene overexpression negatively affected the clinical response to the mAb combination treatment [58]. In another study, Zhu and his team first analyzed patient data of the GO30140 study to define an adequate AFP response cutoff and timepoint, then validated its prognostic value in IMbrave150 patients, and concluded that a ≥75% decrease or ≤10% increase in AFP levels in the first six weeks after treatment initiation correlates with longer OS and PFS, thus representing a potential prognostic biomarker [59].

To date, three matching-adjusted indirect comparisons have been performed, focusing on the efficacy of atezolizumab/bevacizumab versus lenvatinib (plus pembrolizumab), or TARE. Casadei-Gardini et al. applied the comparison to lenvatinib-treated patient data to aggregate IMbrave150 results, and highlighted the superiority of atezolizumab/bevacizumab to lenvatinib, with a hazard ratio (HR) of 0.59 for the OS [60]. Similarly, Jiang et al. exploited the individual data of sorafenib-treated HCC patients and the KEYNOTE 524/IMbrave150 aggregate data, and reported both atezolizumab/bevacizumab and lenvatinib/pembrolizumab superiority to sorafenib. Nonetheless, atezolizumab/bevacizumab, despite being regarded as the first-line immunotherapy for unresectable HCC, showed similar efficacy on OS (HR: 0.71) and PFS (HR: 0.95) as the second-line lenvatinib/pembrolizumab therapeutic regimen [61]. By comparing patient data from the SARAH and aggregate data from the IMbrave150 trials, Agirrezabal et al. concluded that time to deterioration in quality of life after atezolizumab/bevacizumab or TARE treatment does not significantly differ between the two interventions, but still outweighs sorafenib treatment [62].

Except for the IMbrave150 trial, a great number of international study groups have, meanwhile, also performed single- or multi-center clinical trials, with a view to further assessing the safety and efficacy of atezolizumab/bevacizumab, especially in patients with unresectable HCC not meeting the IMbrave150 inclusion criteria. All of these studies have unanimously reached the conclusion that atezolizumab/bevacizumab may also represent a potent later-line therapeutic regime for pretreated patients with unresectable HCC [63,64,65,66,67,68], with Hayakawa et al., however, stating that this mAb combination is more efficient as a first- than as a later-line therapy [69]. Additionally, neither D’Alessio et al. nor Hiraoka et al. could find any disadvantage of the application of atezolizumab/bevacizumab in patients with impaired liver function [21], or HCC classified as beyond up to seven criteria [70].

AFP level, ALBI grade, Child–Pugh score, Neutrophil-to-Lymphocyte ratio, DNA profile, and mutation status, as well as age, all represent confirmed factors predictive of HCC development and progression. Three independent study groups underlined the prognostic value of AFP response at six weeks for the evaluation of atezolizumab/bevacizumab treatment efficacy [63,69,71], while both Iwamoto et al. and Komatsu et al. demonstrated that atezolizumab/bevacizumab can be effective for unresectable HCC, irrespective of the ALBI grade [66,67]. Of note, three research groups more precisely suggested that lower ALBI grades correlate with higher therapeutic efficacy and continuation [72,73,74]. Furthermore, a low Neutrophil-to-Lymphocyte ratio was characterized as a positive predictive factor for survival in atezolizumab/bevacizumab-treated patients with unresectable HCC [75,76,77,78,79], while pretreatment circulating cell-free DNA profiling was found to serve as a biomarker for predicting atezolizumab/bevacizumab therapeutic outcomes [80]. Remarkably, Manzar et al. recently described quick absolute lymphocyte count recovery as a favorable prognosticator of combined treatment with atezolizumab/bevacizumab and radiation therapy [81]. On the contrary, an older patient age or positive *CTNNB1* mutation status did not seem to negatively affect atezolizumab/bevacizumab efficacy and tolerability [82,83,84]. As for sarcopenia, Matsumoto et al. concluded that a decreased skeletal muscle mass index significantly correlated with PFS [85], whereas Toshida et al. could not confirm such an association [86]. Moreover, three studies evaluated the predictive value of radiologic criteria for response to atezolizumab/bevacizumab treatment, and determined the tumor-to-normal liver ratio in the pretreatment Positron Emission Tomography (PET) with 18F-fluorodeoxyglucose, the intensity of hepatobiliary phase of gadoxetic acid-enhanced Magnetic Resonance Imaging (MRI), as well as Choi criteria, as predictors of early progressive disease, OS, and/or PFS in patients with unresectable HCC [87,88,89].

In comparison with lenvatinib as first-line treatment for unresectable HCC, atezolizumab/bevacizumab has also been proven to represent an equal, or even superior therapeutic alternative, in terms of OS, PFS, and treatment-associated adverse events [90,91]. Moreover, the combination of atezolizumab with bevacizumab shows longer PFS than atezolizumab alone in patients with unresectable HCC, given the co-activation of VEGF signaling pathway and tumor immune microenvironment [92,93].

### 2.6. Tremelimumab–Durvalumab

Like Ipilimumab, tremelimumab is another human IgG mAb that prolongs T-cell activation by binding CTLA-4 [94]. Durvalumab is another anti-PD-L1 mAb that promotes antitumor immune-cell-mediated responses [95].

On 21 October 2022, tremelimumab/durvalumab became the most recently FDA-approved mAb combinational treatment regime for adult patients with unresectable HCC, based on the results of the randomized, open-label, multicenter, phase 3 HIMALAYA study [96]. Song et al. obtained patient data from all tremelimumab-containing arms of the Study 22, a phase I/II study that preceded the HIMALAYA study, in order to perform exposure–response analyses of tremelimumab monotherapy or in combination with durvalumab in patients with unresectable HCC. The predicted minimum serum tremelimumab concentration in the novel single-dose regimen of 300 mg tremelimumab in combination with durvalumab was greater than the estimated tremelimumab concentration eliciting half-maximal increases in CD8+Ki67+ T cells, thus proving the substantial and relevant immune response after tremelimumab/durvalumab application in patients with unresectable HCC [97].

**Figure 2:**
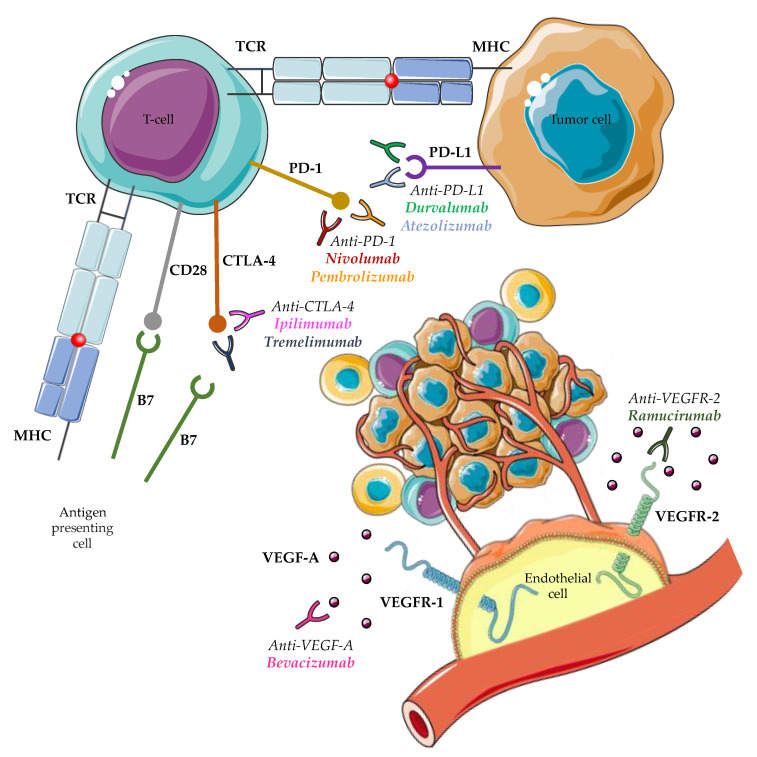
Mechanism of action of FDA-approved monoclonal antibodies for unresectable hepatocellular carcinoma.

Figure 2 summarizes the mechanism of action of the aforementioned FDA-approved mAbs in patients with unresectable HCC.

Table 1 summarizes the application of FDA-approved mAbs in patients with unresectable HCC.

## 3. Discussion

Liver and intrahepatic bile duct cancer represented the sixth most common cause of cancer death in the United States in 2020 [98]. The incidence of HCC, the most frequent primary liver malignancy, still remains high, due to the persistent prevalence of HBV/HCV infections, profuse alcohol consumption, as well as non-alcoholic fatty liver disease (NAFLD) [99,100,101]. Nonetheless, patients with advanced HCC, may nowadays especially profit from targeted agents, which demonstrate a modest but still statistically significant treatment response and survival benefit over conventional chemotherapy [99,102,103,104,105]. Unfortunately, not all HCC patients are potential candidates for targeted therapy, as most immune-checkpoint inhibitors are contraindicated in patients suffering from immune-related or cardiovascular comorbidities [106,107]. Moreover, rare but still life-threatening adverse events may occur, such as the problem of hemorrhagic adverse event risks under bevacizumab treatment [108].

Nivolumab (-ipilimumab), pembrolizumab, and ramucirumab are FDA-approved second-line mAbs for unresectable HCC in patients pretreated with sorafenib, the standard of care systemic TKI for advanced HCC since 2007 [109]. All three agents show a relative efficacy in pretreated HCC patients with an unsatisfactory response to sorafenib treatment, with tumor characteristics, disease severity, as well as pretreatment status, representing useful predictive factors, which could help clinicians select suitable candidates, and spare potentially inadequate patients from unnecessary toxicity. In this context, nivolumab undoubtedly represents the mAb with the most identified response predictors: the better the clinical condition of the patient and the liver function, the more effective the response to nivolumab therapy. Additionally, pembrolizumab’s efficacy has been sufficiently studied in combination with lenvatinib and/or TACE treatment, thus paving the way for the development of further potential combinational regimes that could revolutionize the therapy of unresectable HCC. Regrettably, ramucirumab does not seem to exert auspicious anti-cancer effects in patients with unresectable HCC.

On the contrary, the combination of atezolizumab with bevacizumab is superior to sorafenib treatment, and thus is suggested as the first-line therapeutic regime for patients with preserved liver function over sorafenib or lenvatinib [110]. Of note, atezolizumab/bevacizumab does not, nevertheless, seem to be superior to second-line treatment approaches including lenvatinib/pembrolizumab or TARE. Importantly, atezolizumab/bevacizumab might serve as a potent first- or later-line agent in patients not fulfilling the IMbrave150 inclusion criteria. Given its leading role in modern HCC therapy, numerous study groups have, to date, examined and determined the prognostic role of molecular, laboratory, clinical, and radiological features for ORR, OS, and PFS, thus allowing for the establishment of patient selection criteria and useful biomarkers. The ALBI grade, which along with the Child–Pugh score system, has been integrated into the guidelines for the management of HCC as a liver assessment tool [111], is the most extensively studied parameter, with low ALBI grades correlating with higher therapeutic efficacies. Despite its promising effects, atezolizumab/bevacizumab does not, however, seem to represent a cost-effective treatment option, a disadvantage that undoubtedly needs to be taken into consideration in times of financial uncertainty.

Another noteworthy aspect is the problem of HCC patients with a suboptimal liver function such as those with Child–Pugh B class liver functions for whom, currently, there are few available data on the safety profile, which are almost exclusively related to sorafenib therapy [112].

Interestingly, quantitative systems pharmacology models are actively being used in both the pharma industry and academic research, and have the potential to design clinical trials. Recently, the first-ever model for HCC was published, which predicts the outcome of CheckMate 040, hence facilitating clinical trial decision-making and drug approval processes [113].

Last but not least, even though immune checkpoint inhibitors seem to have finally found their role in HCC as part of combinatorial strategies, several questions still remain unanswered. Among these, the lack of validated biomarkers of response represents an important issue, since only a proportion of HCC patients benefit from immunotherapy. Based on these premises, a greater understanding of the role of potential biomarkers including PD-L1 expression, tumor mutational burden (TMB), microsatellite instability (MSI) status, gut microbiota, and several others, is fundamental [114,115].

## 4. Conclusions

Taken altogether, the current review summarizes the results of all relevant clinical trials on FDA-approved mAbs, and provides a useful update on mAb-based targeted treatment for patients with unresectable HCC. Even though several researchers have already attempted to define the most appropriate therapeutic regime for each HCC patient subgroup, clinical trials on HCC immunotherapy widely differed in terms of drugs, patients, designs, terms of study phases, and inconsistent clinical outcomes. Future trials need to focus more on the comparison of the effects of different agents, and to further investigate the possibility of combinational treatments, in order to evaluate the potential additive effect of current individual modest therapies. Future research is also required in order to identify tools able to predict treatment response, both biological and clinical.

## Figures and Tables

**Table 1:** The use of FDA-approved monoclonal antibodies in patients with unresectable hepatocellular carcinoma.

Agent	Action Mechanism	Response Predictors	Comparison with Other Treatments	References
Nivolumab	PD-1 inhibitor	Child–Pugh score CLIP score AFP level ALBI grade Number and size of lesions Vascular invasion Immune-related adverse events Prior TARE	Second-line treatment alternative for sorafenib-pretreated HCC	[23,24,25,27,28]
Pembrolizumab	PD-1 inhibitor	TGF-β level Microsatellite instability	Second-line treatment alternative for sorafenib-pretreated HCC Synergy with lenvatinib and TACE	[30,31,33,35,36,37,38,39]
Ramucirumab	VEGFR2 inhibitor	AFP level Treatment response to prior TKI therapy	Second-line treatment alternative for sorafenib-pretreated HCC with AFP levels ≥ 400 ng/mL	[41,42,44]
Nivolumab+ Ipilimumab	PD-1 inhibitor Anti-CTLA-4	SBRT	Second-line treatment alternative for sorafenib-pretreated HCC	[46]
Atezolizumab+ Bevacizumab	Anti-PD-L1 Anti-VEGF	Pre-existing immunity PD-L1 mRNA level Myeloid inflammatory response CD8+/regulatory T-cell count VEGFR2 expression Regulatory-to effector-T-cell ratio Oncofetal gene expression AFP level Liver function ALBI grade Neutrophil-to-Lymphocyte ratio Circulating cell-free DNA profiling Absolute lymphocyte count recovery *CTNNB1* mutation Patient age Sarcopenia Radiologic criteria	Superior to sorafenib treatment Equal to lenvatinib/pembrolizumab, and TARE treatment	[21,48,58,59,60,61,62,63,66,67,69,70,71,72,73,74,75,76,77,78,79,80,81,82,83,84,85,86,87,88,89,90,91]
Tremelimumab+Durvalumab	Anti-CTLA-4 Anti-PD-L1	Serum tremelimumab concentration	Superior to sorafenib treatment	[97]

PD-1: Programmed cell death protein 1; CLIP: Cancer of the liver Italian program; AFP: Alpha-fetoprotein; ALBI: Albumin-bilirubin; TARE: Transarterial radioembolization; HCC: Hepatocellular carcinoma; TGF-β: Transforming growth factor beta; VEGFR2: Vascular endothelial growth factor receptor 2; TKI: Tyrosine kinase inhibitor; CTLA-4: Cytotoxic T-lymphocyte antigen-4; SBRT: Stereotactic body radiation therapy; VEGF: Vascular endothelial growth factor.

## Data Availability

Not applicable.
